# Malignant and Non-Malignant Causes of Hypercalcemia: A Retrospective Study at a Tertiary Care Hospital in Pakistan

**DOI:** 10.7759/cureus.15845

**Published:** 2021-06-22

**Authors:** Sabiha Banu, Sumera Batool, Saadia Sattar, Muhammad Q Masood

**Affiliations:** 1 Endocrinology and Diabetes, Aga Khan University Hospital, Karachi, PAK; 2 Medicine, Aga Khan University Hospital, Karachi, PAK; 3 Medicine, Endocrinology, Aga Khan University Hospital, Karachi, PAK

**Keywords:** hypercalcemia, malignancy, hyperparathyroidism, vitamin d toxicity, mortality

## Abstract

Background: Hypercalcemia is a common electrolyte abnormality presenting with a variety of symptoms. The common causes are primary hyperparathyroidism and malignancy associated with hypercalcemia. However, iatrogenic hypercalcemia with the overzealous use of vitamin D has now emerged as another important cause of hypercalcemia over the past decade.

Objective: This study aims to evaluate the causes of hypercalcemia, management strategies, and outcomes in patients admitted with hypercalcemia in a tertiary care hospital.

Method: It is a retrospective study done at Aga Khan University Hospital (AKUH), Karachi after taking approval from the ethical review committee (ERC). Data were gathered about all patients admitted from 1st^ ^January 2008 to 31st^ ^December 2018. A total of 1142 patients were included in the study and their calcium levels were noted. Along with demographic details, data pertaining to their diagnosis and all investigations done to evaluate the causes of hypercalcemia were noted in a pre-defined questionnaire.

Results: A total of 814 patients having hypercalcemia were included in the final analysis and their mean age was 60.8 ± 14.1 years. Male and female patients were 45.4% and 54.6%, respectively, and their mean hospital stay was 6.2 ± 5.8 days. The most common cause of hypercalcemia was malignant solid tumors (49.1%), followed by hematological malignancy (16.5%), hyperparathyroidism (10.9%), definite vitamin D toxicity (8%), chronic kidney disease (4.9%), chronic granulomatous diseases (4.7%), and probable vitamin D toxicity (3.5%). The oral cavity carcinoma (17.7%) was the most common solid tumor associated with hypercalcemia. Amongst hematological malignancy, multiple myeloma (14.4%) was the most common one. Out of 814 patients admitted with hypercalcemia, 601 (74%) patients recovered from hypercalcemia, while mortality was observed in 129 (16%) patients. Of those who expired, 110 (85.3%) had malignancy either solid tumor or hematological.

Conclusion: Malignancy is the most common cause of hypercalcemia in admitted patients. The knowledge of hypercalcemia’s causes is of great importance so that targeted investigations can be done. Not only will it minimize the cost burden and shorten the hospitalization of patients; it will also help the physicians to decide the appropriate management accordingly. Moreover, vitamin D toxicity was also observed in a significant number of patients which highlights the common practice of using higher doses of vitamin D by physicians.

## Introduction

Hypercalcemia is labeled when serum calcium levels are above the laboratory-specific reference range, i.e. albumin corrected serum calcium >10.5 mg/dL or ionized calcium level >5.52 mg/dL [1]. It is a frequently encountered electrolyte abnormality and can present with a wide range of symptoms and signs. It can also be detected incidentally in asymptomatic patients. Causes of hypercalcemia can be broadly divided into parathyroid hormone (PTH) dependent and PTH-independent etiologies. The two most frequently encountered etiologies are primary hyperparathyroidism and hypercalcemia secondary to malignancy. These two diagnoses contribute to 80%-90% of all cases of hypercalcemia [1-2]. In the outpatient setting, the predominant cause of hypercalcemia is hyperparathyroidism. In hospitalized patients, it is typically malignancy [3]. However, other causes like vitamin D toxicity and milk alkali syndrome, which were rarely encountered in the past, are now being seen more frequently. In one study vitamin D toxicity was the second common cause of hypercalcemia after primary hyperparathyroidism [4].

The majority of studies are done to evaluate the presence of hypercalcemia in different diseases. The prevalence of hypercalcemia in solid cancers was found to be 21.92% [5]. Similarly, 51.2% of multiple myeloma patients had hypercalcemia [6]. However, the current literature is scarce for different disorders, including both malignant and non-malignant contributing to hypercalcemia.

The parathyroid hormone plays an important role in the mobilization of calcium from bones, reabsorption of calcium from the kidney, and activation of alpha one hydroxylase leading to the formation of the activated form of vitamin D (calcitriol). Activated vitamin D is required for the reabsorption of calcium from the renal tubules and intestine [6]. Therefore, hypercalcemia is either due to increased calcium reabsorption from the small intestine, renal tubules, or increased bone resorption. There are four causes of malignancy-associated hypercalcemia: (1) increased production of PTH-related peptide (PTHrP) by tumor cells (termed as humoral hypercalcemia of malignancy); (2) bone dissolution by metastasis from a tumor or multiple myeloma due to release of local PTHrP and osteolysis; and (3) excess PTH production by cancerous cells (ectopic PTH production or from parathyroid carcinoma) which is rare [1, 7].

In this study, we will discuss the frequency of different disorders contributing to hypercalcemia. The knowledge of these disorders and their frequency in our region is a need of the present time for the clinicians. It will help to advise appropriately cost-effective investigations and management options and prevent iatrogenic conditions like vitamin D toxicity. We have also looked for the management opted for hypercalcemia and its outcome in our study participants.

## Materials and methods

This is a retrospective study analyzing all the patients admitted to the hospital from 1st January 2008 to 31st December 2018 who had hypercalcemia. It is done in Aga Khan University Hospital (AKUH), Karachi, Pakistan which is a tertiary care hospital. After approval from the ethical review committee (ERC) of AKUH, a list of 1142 patients was generated by the health information and management system (HIMS) department. All the patients who had serum calcium levels (either albumin corrected or ionized calcium) above the normal range were included in the study. A normal range for albumin corrected calcium level was 8.5-10.5 mg/dL and ionized calcium level was 4.8-5.52 mg/dL. At least two abnormal readings were required to label it as a case of hypercalcemia. The demographic details were noted, and the electronic health record was reviewed for the investigations done for evaluation of the etiology of hypercalcemia. This included serum phosphate, serum creatinine, erythrocyte sedimentation rate (ESR), parathyroid hormone (PTH) levels, and 1,25OH vitamin D levels. We also reviewed histopathology, radiological records, and discharge summaries for the diagnosis of disorders like granulomatous disorders, multiple myeloma, or any malignancy. The diagnosis of primary hyperparathyroidism was made when serum PTH levels were raised or inappropriately normal in the presence of hypercalcemia. Definite vitamin D toxicity was considered when the 1,25OH vitamin D levels were > 150 ng/mL [8] while the level of 80-149 ng/mL in the absence of any other cause of hypercalcemia was labeled as probable vitamin D toxicity.

Due to the fact that serum PTHrP and 1,25OH vitamin D levels were not available in our institute, we do not have data about these laboratory values in our study participants. After establishing the etiology of hypercalcemia, we looked for the hospital course of these patients, management strategies opted for hypercalcemia and the response in terms of normalizing serum calcium level. The outcome was noted as discharge or death of the patient. Despite thorough investigations and the extensive review of the hospital record, the patients in whom the cause of hypercalcemia was not obvious were labeled as hypercalcemia of unknown cause. All these data were recorded on a pre-defined questionnaire.

## Results

Records of 1142 patients admitted to AKUH with hypercalcemia between January 2008 and December 2018 were reviewed as per the inclusion criteria. Out of 1142 patients, 328 (28.7%) patients were excluded because of hypocalcemia (15 patients), normocalcemia (112 patients), transient hypercalcemia (61 patients), iatrogenic hypercalcemia (3 patients), idiopathic (48 patients), and incomplete workup (89 patients). The electronic health records of the remaining 814 (71.3%) patients were reviewed and analyzed.

Out of 814 hypercalcemia patients, 370 (45.4%) were males and 444 (54.6%) were females with the mean age of 60.8 ± 14.1 years. The mean hospital length of stay was 6.2 ± 5.8 days. The length of stay was less than four days in 398 (48.9%) patients and more than equal to four days in 51.1% of patients. Patients with different levels of 1,25OH vitamin D and PTH are characterized in Table 1. Serum creatinine was 2.1 1.8 on admission while on discharge it was 1.6 1.5. ESR and serum protein electrophoresis was checked in 236 (29.3 %) and 245 (30.2%) patients, respectively (Table 1). The length of stay in patients with probable toxicity was 6.7 ± 5.6 days and in patients with definitive vitamin D toxicity was 6.0 ± 3.7 days. During the hospital stay, hypercalcemia was managed through the administration of bisphosphonates (77.5%), steroids (38.8%), and calcitonin (17.9%) in patients (Table 1).

**Table 1:** Demographic characteristics and laboratory data of the study participants (n=814). *mean±SD LAMA, left against medical advice; PTH, parathyroid hormone; SD, standard deviation Note: Normal PTH is 16-87 pg/mL

Characteristics	n (%)
Age (in years)*	60.8 ± 14.1
Age by gender*	
Male	60.7 ± 14.4
Female	60.9 ± 13.9
Gender	
Male	370 (45.4)
Female	444 (54.6)
Mean length of stay*	6.2 ± 5.8
Length of stay	
<4 days	398 (48.9)
≥4 days	416 (51.1)
Number of patients in whom PTH checked	380 (46.6)
Low PTH level	227 (59.7)
Normal PTH level	48 (12.6)
High PTH level	105 (27.6)
Serum calcium*	13.2 ± 1.9 mg/dl
Serum protein electrophoresis	245 (30.2)
Erythrocyte sedimentation rate	236 (29.3)
Creatinine on admission*	2.1 ± 1.8 mg/dl
Creatinine on discharge*	1.6 ± 1.5 mg/dl
Duration of normalization of calcium	
<3 days	182 (22.4)
3-5 days	241 (29.6)
>5-10 days	100 (12.3)
>10 days	18 (2.2)
Not normalized	273 (33.5)
25 OH Vitamin D level	
<30 mg/dL	180 (22.1)
30-79 mg/dL	96 (11.8)
80-149 mg/dL	42 (5.2)
>150 mg/dL	61 (7.5)
Level not checked	435 (53.4)
Medications administered	
Bisphosphonates	631 (77.5)
Steroids	316 (38.8)
Calcitonin	146 (17.9)
Outcome	
Recovered from hypercalcemia	601 (73.8)
Expired	129 (15.8)
LAMA	59 (7.3)
Parathyroidectomy	25 (3.1)

Calcium levels were not normalized in 273 (33.5%) patients despite all standard treatment options whereas, 22.4% of patient’s calcium levels were normalized in less than three days, 29.6% in three to five days, 12.3% in five to ten days, and 2.2% were normalized in more than 10 days (Table 1). Out of 273 (33.5%) patients in whom calcium level was not normalized, 182 (66%) patients were found to have malignancy (146 patients had solid tumor malignancy and 36 patients had hematological malignancy).

The most common cause of hypercalcemia was solid tumor malignancy (49.1%) accompanied by hematological malignancy (16.5%), hyperparathyroidism (10.9%), definite vitamin D toxicity (8%), chronic kidney disease (4.9%), chronic granulomatous disease (4.7%), and probable vitamin D toxicity (3.5%) (Table 2). 

**Table 2:** Causes of hypercalcemia (n=814).

Diagnosis	n (%)
Solid tumor malignancy	400 (49.1)
Hematological malignancy	133 (16.5)
Hyperparathyroidism	89 (10.9)
Definite vitamin D toxicity	65 (8.0)
Chronic kidney disease	40 (4.9)
Chronic granulomatous diseases	38 (4.7)
Probable vitamin D toxicity	28 (3.4)
Hyperparathyroidism and solid tumor malignancy	5 (0.6)
Vitamin D toxicity and solid tumor	4 (0.5)
Vitamin D toxicity and hyperparathyroidism	4 (0.5)
Vitamin D toxicity and hematological malignancy	4 (0.5)
Hyperparathyroidism and chronic granulomatous disease	2 (0.2)
Vitamin D toxicity and chronic granulomatous disease	1 (0.1)
Hyperparathyroidism and hematological malignancy	1 (0.1)

Among solid tumor malignancy, oral cavity (17.7%) was the most common followed by breast carcinoma (14.7%), lung carcinoma (8.8%), and 5.8% of patients suffered metastatic tumor with unknown primary. In patients with hematological malignancy, the most common malignancy was multiple myeloma (14.4%) followed by non-Hodgkin lymphoma (7.4%), Hodgkin lymphoma (1.5%), chronic lymphocytic leukemia (CLL) (0.9%), chronic myeloid leukemia (CML) (0.2%), and acute myeloid leukemia (AML) (0.2%) (Table 3).

**Table 3:** Sub-diagnosis in patients with hypercalcemia secondary to malignancy (n=571).

Sub-diagnosis	n (%)
Oral cavity carcinoma	101 (17.7)
Breast carcinoma	84 (14.7)
Multiple myeloma	82 (14.4)
Lung carcinoma	50 (8.8)
Non-Hodgkin lymphoma	42 (7.4)
Metastatic tumor	33 (5.8)
Hepatocellular carcinoma	30 (5.2)
Renal cell carcinoma	19 (3.3)
Malignant neoplasm of esophagus	14 (2.9)
Ovarian carcinoma	13 (2.3)
Extra pulmonary tuberculosis	13 (2.4)
Pulmonary tuberculosis	12 (2.1)
Cholangiocarcinoma	11 (1.9)
Colorectal carcinoma	10 (1.9)
Bladder cancer	8 (1.5)
Hodgkin lymphoma	8 (1.5)
Pancreatic tumor	7 (1.2)
Neuroendocrine tumor	5 (0.9)
Chronic lymphocytic leukemia	5 (0.9)
Sarcoma	4 (0.7)
Squamous cell carcinoma	4 (0.7)
Prostate cancer	4 (0.7)
Endometrial cancer	3 (0.5)
Cervical tumor	2 (0.4)
Stomach carcinoma	1 (0.2)
Malignant neoplasm of uterine adnexa	1 (0.2)
Papillary thyroid carcinoma	1 (0.2)
Chronic myeloid leukemia	1 (0.2)
Brain carcinoma	1 (0.2)
Acute myeloid leukemia	1 (0.2)

Out of 814 patients, 601 (74%) patients recovered from hypercalcemia during the hospital stay and were sent back home, 59 (7.3%) patients left against medical advice (LAMA), 25 (3%) underwent parathyroidectomy, and 129 (16%) patients expired (Table 1). Among 129 expired patients, 93 (72%) patients were found to have a malignant solid tumor and 18 (14%) patients died because of hematological malignancy. Causes of mortality in other patients were hyperparathyroidism in six (4.6%) patients, vitamin D toxicity in 4 (3.1%) patients, chronic kidney disease in 4 (3.1%) patients, and chronic granulomatous disease in 4 (3.1%) patients. Among expired patients, serum calcium levels ranged from 11 to 19.9 mg/dL. An important point to note is that among 129 expired patients, 44 patients had a serum calcium level of ≥ 14 mg/dL.

Out of 40 patients with chronic kidney disease, 14 (35%) patients had low PTH levels, 11 (27.5%) had normal PTH levels, and 15 (37.5%) had high PTH levels. Out of 814 patients with hypercalcemia, 13 patients underwent hemodialysis due to their underlying end stage renal disease (ESRD), but not due to hypercalcemia. Reasons of hospitalization in our hypercalcemia patients were drowsiness, reduced oral intake requiring IV fluids administration, vomiting, and extreme lethargy. 

## Discussion

The most common cause of hypercalcemia in our study was found to be malignant solid tumor followed by hematological malignancy. In one study published in 2018, malignancy was the leading cause of hypercalcemia in hospitalized patients (54%-65%) [1]. Hypercalcemia may occur in up to 30% of patients diagnosed with malignancy, and this carries a grave prognosis [9]. Hypercalcemia arisen from malignancy is especially associated with advanced malignancy.

The most prevalent malignant solid tumor in our study was oral cavity carcinoma (17.7%) followed by breast (14.7%) and lung carcinomas (8.8%). The increased incidence of oral cavity carcinoma in our population is because of the increased use of tobacco and betel nuts in all age groups and in both genders. In one study, the common tumors associated with humoral hypercalcemia of malignancy were squamous cell carcinomas of the lung, head and neck cancers, esophagus, skin, cervix, breast, kidney, prostate, or bladder cancers [9]. According to World Health Organization (WHO), there will be a marked increase in oral malignancies, especially in South Asian countries where their incidence is the highest among all urban cancer registries, in contrast to data worldwide [10]. In Western countries, alcohol and tobacco are known as the contributing risk factors in oral cancer [11], but in Asian countries, the main carcinogens are betel nuts and their substitutes. According to the Shaukat Khanum cancer registry Lahore in Pakistan, oral cavity tumor is the second most common malignancy after lung cancer in males and breast cancer in females [12]. In contrast, we found oral cavity tumors as the most common tumor and malignant solid tumors as the most common cause of hypercalcemia in our patient population. The main reason for malignant disorders being on the top of the list is our patient population, as we have included only patients admitted in the hospital with hypercalcemia. Malignancy is associated with rapid onset and severe hypercalcemia, often requiring in-hospital management. While the disorders like primary hyperparathyroidism are mainly diagnosed and managed on an outpatient basis. 

The hematological malignancy was the second most common cause of hypercalcemia in our study. The most common malignancies contributing in this group were multiple myeloma in 82 patients (14.4%), followed by Non-Hodgkin lymphoma in 42 patients (7.4%). In one study published in the *Journal of Leukemia and Lymphoma*, the presence of hypercalcemia in patients with newly diagnosed diffuse large B cell lymphoma was found to be 18% [13]. In a study published in *Annals of Intensive Care* in 2019, the hematological malignancies were the contributing cause of hypercalcemia in 44.3% of patients [14].

Hyperparathyroidism is the third common cause accounting for 10.9% of hypercalcemia in our study. With widespread testing for calcium levels being done these days, primary hyperparathyroidism is now commonly diagnosed in an asymptomatic phase. It is the most frequent etiology of hypercalcemia in the outpatient population [15]. In our study 89 (10.9%) patients were found to have hyperparathyroidism in which 52 patients were found to have single parathyroid adenoma on imaging (ultrasound neck and sestamibi scan). Out of these 52 patients, 25 patients underwent parathyroidectomy at our hospital. On histopathological report 23 patients were found to have parathyroid adenoma, one patient had parathyroid hyperplasia and one patient had parathyroid carcinoma. On presentation, the patient with parathyroid carcinoma had a calcium level of 15 mg/dL and PTH was 2237 pg/mL and it took nine days for his calcium to get normalized during the hospital stay. In another study of 103 patients, localization of parathyroid adenoma was made via ultrasound neck in 64.1% and via sestamibi scan in 76.2% of patients. Out of 103 patients, 79 patients underwent parathyroidectomy. On histopathological report parathyroid adenoma was found in 67 (84.8%) patients, hyperplasia was found in five patients, and parathyroid carcinoma was found in two patients [16]. In our study, one patient was found to have primary hyperparathyroidism along with hematological malignancy. The coexistence of primary hyperparathyroidism and multiple myeloma as the etiology of hypercalcemia is rare in the literature; only 30 cases are there so far [17].

Vitamin D toxicity is the fourth common contributor of hypercalcemia in our study. Vitamin D toxicity is diagnosed by serum levels of vitamin D3 above 150 ng/mL. It is characterized by severe hypercalcemia and low or undetectable PTH hormone levels [18]. In our study, the exact amount of vitamin D taken by patients could not be retrieved because of the retrospective nature of the study. We observed definite vitamin D toxicity in 65 patients with serum vitamin D3 levels above 150 pg/mL and 28 patients had probable vitamin D toxicity with serum levels between 80 and 149 pg/mL. Out of 65 patients with definite vitamin D toxicity, calcium was not normalized in 25, despite using multiple treatment options. While in the probable vitamin D toxicity group, out of 28 patients, calcium was not normalized in four till the time of discharge. The treatment options opted were good hydration, IV steroids, and bisphosphonate therapy.

In our study, the treatment options used to treat hypercalcemia were bisphosphonates (77.5%) followed by steroids (38.8%) and calcitonin (17.9%) in order of frequency. Denosumab was not available at the time of our study period. In our study in the hematological malignancy group, 69 patients (51.8%) received steroids. This data is comparable to one study published in the *Annals of Intensive Care* in November 2019 in which out of 131 patients with hypercalcemia, 124 patients (94.6%) received IV fluids, 103 (78.6%) received bisphosphonates particularly those with solid tumor and hematological malignancies, 65 (50%) patients received corticosteroids, 12 patients (9%) received calcitonin, and none of the patients received denosumab. Out of those patients who received corticosteroids 84.4% of patients had a diagnosis of hematological malignancy [14]. In another study, bisphosphonates were found to be very effective and safe in patients with hypercalcemia of malignancy [19-20].

The workup of hypercalcemia was incomplete in 89 (8.7%) patients. Because of the retrospective nature of the study, it was difficult to label a definite diagnosis in these patients. Transient hypercalcemia was labeled where serum calcium levels normalized within the first 24 hours and it was observed in 61 patients (6%). In 48 (4.73%) patients no cause of hypercalcemia was found, despite the extensive standard workup of hypercalcemia. Other causes of hypercalcemia in our study include chronic kidney disease in 40 patients (4.9%) and chronic granulomatous diseases in 38 patients (4.7%) (including mainly tuberculosis and sarcoidosis).

The majority of the participants 73.8% (601) had normalization of serum calcium levels and were discharged home, while 15.8% (129) were expired either due to complications related to hypercalcemia or primary disorder. The majority of the patients who expired had primary disorder as malignancy, i.e. 93 patients had solid tumor malignancy and 18 patients had hematological malignancy.

## Conclusions

Appropriate identification of underlying diagnosis and treatment of hypercalcemia is very essential in limiting morbidity and mortality associated with this entity, prolonged length of hospital stay, and economical burden on patient’s family (secondary to extensive workup and length of hospital stay). History of vitamin D intake should be taken very vigilantly. Physicians should avoid overzealous replacement of vitamin D as it is the need of time to avoid vitamin D toxicity; in order to do so, they should review recommendations from the guidelines of the Endocrine Society in the management of vitamin D deficiency.
